# Approaching person-centered clinical practice: A cluster analysis of older inpatients utilizing the measurements of intrinsic capacity

**DOI:** 10.3389/fpubh.2022.1045421

**Published:** 2022-11-11

**Authors:** Wenbin Wu, Liang Sun, Hong Li, Jie Zhang, Ji Shen, Jing Li, Qi Zhou

**Affiliations:** ^1^Department of Geriatrics, Beijing Hospital, National Center of Gerontology, Institute of Geriatric Medicine, Chinese Academy of Medical Sciences, Beijing, China; ^2^The Key Laboratory of Geriatrics, National Center of Gerontology of National Health Commission, Beijing Institute of Geriatrics, Institute of Geriatric Medicine, Beijing Hospital, Chinese Academy of Medical Sciences, Beijing, China

**Keywords:** intrinsic capacity, hospitalized older adults, clinical practice, unsupervised classification, healthcare

## Abstract

**Background:**

Maintaining the intrinsic capacity (IC) of older inpatients is a novel view in providing person-centered treatments in clinical practice. Uncertainty remains regarding the primary nature of IC among older hospitalized patients.

**Objectives:**

We aimed to understand the status of IC among older inpatients by a cluster analysis based on IC measurements.

**Methods:**

This is a cross-sectional study conducted in the geriatric department of Beijing Hospital in China. Older inpatients who were older than 60 years and who underwent comprehensive geriatric assessments were included. The inpatients were classified into subgroups based on 13 measurements of IC according to unsupervised methods (K-means cluster analysis and t-SNE). Subgroup differences were investigated for domains of IC, age, sex, frailty, activities of daily living, and falls.

**Results:**

A total of 909 inpatients with a mean age of 76.6 years were included. Almost 98% of the inpatients showed IC impairment. Locomotion impairment was the most prevalent problem (91.1%), followed by sensory impairment (61.4%), psychological impairment (57.3%), cognition decline (30.7%), and vitality problem (29.2%). A total of five clusters were obtained by classification: Cluster 1 (56.6% of the participants) showed high IC with fair impairment of locomotion and vision; clusters 2 and 3 (37.8 % of the participants) had additional impairment of sleep in the psychological domain; clusters 4 and 5 (5.6% of the participants) represented a severe loss of all the IC domains; and clusters 1–5 showed a gradual decline in the IC score and were significantly associated with increased age, frailty, decreased activities of daily living, and falls. Significant correlations among the domains were observed; the locomotion domain showed the strongest links to the others in network analysis.

**Conclusions:**

Great declines in IC and disparities between IC domains were found in older inpatients. IC-based primary assessment and classification enabled us to identify the variation of functional abilities among the older inpatients, which is pivotal for designing integrated treatment or care models in clinical practice.

## Introduction

Fostering and maintaining functional ability should be a lifelong pursuit in healthy aging (1). To achieve this goal, the World Health Organization (WHO) has reframed health and healthcare for older adults by proposing the concept of intrinsic capacity (IC) and guidelines of integrated care for older people (ICOPE) (2). IC is defined as the set of physical and mental capacities of an individual covering five domains, namely, locomotion, cognition, sensory, vitality, and psychological domains. Previous studies showed that the domains of IC (3–7) as well as the total score of all domains (5, 6) were associated with self-care, frailty, living dependency, hospitalization, and mortality among the older population. These findings not only highlighted the importance of IC as a useful tool to detect, prevent, and delay the onset of disabilities but also accelerated the shift from a disease-based approach to a person-centered approach in clinical practice.

The concept of IC, defined by the WHO to promote healthy aging, greatly contributes to clinical practice; however, it has been largely underutilized to date (8). Primary studies on IC were usually performed in the community, while the level of IC maintained by the older inpatients or outpatients is scarcely known. To our best knowledge, only two studies involving about 500 inpatients assessed the primary characteristics of IC (9, 10). Moreover, significant loss of functional capacities after hospitalization was common for older adults; adverse outcomes such as readmission and mortality after discharge were also associated with IC (9–12). Thus, estimating older adults' IC in the hospital is essential to plan and implement interventions to maintain their functional ability.

Consensus on how to assess the different dimensions of IC has not been reached yet; also, the relationship between the domains of IC is not clear (8, 13), which is important because it affects how we understand and use IC in clinical practice. A total of three modes were commonly used to quantify IC: a sum of *z*-scores of each domain divided by the number of domains or a summed total score (3, 13), a single use of each domain (14), and a cluster analysis (6). The variation in the scoring system reflects the conceptual obscurity surrounding the construct of IC. Therefore, more systematic assessments, such as investigating the correlation between domains and performing dimensionality reduction, are necessary to understand the conceptual framework of IC.

The current study aimed to understand the functional abilities of older Chinese inpatients by assessing the primary features of IC, classifying them into clusters, and exploring the relationship between the clusters and age, sex, activities of daily living, frailty, and falls.

## Materials and methods

### Participants and data collection

This is a cross-sectional study of comprehensive geriatric assessment (CGA) for elderly inpatients at Beijing Hospital in China. All the participants aged ≥60 years were enrolled in the department of geriatrics from 1 May 2020 to 1 May 2022, but those with incomplete measurements of IC were excluded. Data for the following variables were collected: age, sex, body mass index (BMI), Barthel index for basic activities of daily living (BADL), Lawton instrumental activities of daily living score (IADL), fried frailty phenotype (FFP), Clinical Frailty Scale (CFS), number of falls in the past year, and measurements of the five domains of intrinsic capacity. A total of 909 participants were finally assessed. This study was approved by the Medical Ethics Committee of Beijing Hospital (2021BJYYBC-173-01), and endorsed informed consent was obtained from each participant.

### Measurements of IC

A total of five IC domains were defined according to 13 measurements. A domain was defined as “impaired” if one or more of its measurements of functions was considered declined or impaired.

The locomotion domain was measured using a Short Physical Performance Battery (SPPB) that included a 4-m walking speed test at the usual pace, a hierarchical test of standing balance, and five repetitive chair rise test (15). A walking speed of <1.0 m/s, more than 12 s to complete the five chair rises, or (16) a total SPPB score of <10 was considered abnormal. Grip strength was also used to represent the locomotion domain. It was measured three times using a handheld dynamometer with each hand. An average score was calculated using the data from the dominant hand. The grip strength test was adjusted by sex; a grip strength of <28 and <18 kg for male and female patients was considered abnormal, respectively.

The vitality domain was measured by low BMI (<18.5 kg/m^2^), unintentional weight loss (≥4.5 kg in the last 3 months), and a Chinese version of the Eat-10 test for swallowing problem measurement (abnormal, score ≥ 3) (16, 17).

The cognition domain was measured by a Mini-Mental State Examination (abnormal, score < 24) (18).

The psychological domain was measured by self-reported depressive symptoms (SDSs), sleep quality, and self-reported satisfaction with life (SLA). SDS was determined by two questions: “1. Over the past 2 weeks, have you been bothered by feeling down, depressed, or hopeless? 2. Do you feel little interest or pleasure in doing things?”. A Chinese version of Athens Insomnia Scale (AIS) was adopted to measure the sleep quality in the past month (abnormal, score > 6) (19, 20). SLA was assessed by one question: “In general, how do you feel about your life? Please score the feeling with numbers from 1 to 10, which represents from very bad to very good, respectively”. The patient with a score of <5 was considered unsatisfied with their life.

The sensory domain included hearing and vision. Hearing was considered adequate when the older person did not report “hearing problems or deafness”, and the interviewer did not identify the person as profoundly deaf. Similarly, the vision was considered normal when the older person did not report an “eye problem” that interfered with their activities to some extent, and the interviewers did not find the older person functionally blind.

The IC score was estimated as follows: a higher score indicated a larger amount of functional capacity reserved for older patients. The IC score was constructed as follows: a decline in each measurement was calculated as 0 or otherwise as 1; each domain score was defined by the mean score of all measurements in this domain; the overall IC score was determined by adding the scores of the five domains.

### Assessment of frailty, disability, and dependence, and falls

FFP and CFS were used to assess frailty. FFP included five items: weakness, slowness, exhaustion, weight loss, and low activity (21). Weakness was determined by decreased grip strength adjusted for sex and BMI (22). Slowness was determined by a reduced walking speed according to a 4-m walk test adjusted for sex and height (23). Exhaustion was indicated by responses to questions of the Center for Epidemiological Studies Depression Scale: “I felt that everything I did was an effort” or “I couldn't get going” (24). Weight loss was defined as a self-reported unintentional weight loss of 3 kg in the past 6 months or having a BMI of 18.5 kg/m^2^. Low activity was defined as self-reported exercise for <3 h/week over the past 12 months (25). Individuals with impairments in over two items were defined as physically frail. For the CFS, the physician scored patients from 1 (very fit) to 7 (severe frailty or more) according to his own judgement. Older people who scored over 3 points were considered clinically frail (26).

Basic and instrumental activities of daily living were determined through the assessment of the performance of older persons on each item of BADL (27) and IADL (28) as independent, partially dependent, and completely dependent and adding the scores, which ranged from 0 to 100 for BADL and 0 to 8 for IADL, respectively. Lower scores indicated worse basic or instrumental living performance.

Inpatients or their families recalled the number of falls in the past year according to the question “how many time the patient has fallen in the past year?”.

### Unsupervised classification and visualization

Two unsupervised methods, k-means and t-distributed stochastic neighbor embedding (t-SNE), were used to classify the participants. Specifically, clusters were formed based on the 13 variables that were used to build the IC score. The raw score of the measurements were used in the k-means algorithm. Then, t-SNE, a non-linear dimension reduction technique, was used to model high-dimensional data into a two-dimensional space to show the pairwise distance among the participants and visualize the clusters formed by k-means. The classified clusters were interpreted based on the basis of the characteristics of the IC domains. k-means and t-SNE were performed using basic packages and “Rtsne” packages, respectively, in R language.

### Statistical analysis

All the analyses and plots were performed using the R language (R x64 V4.1.2). A chi-square test was used to analyze the differences in the prevalence of ADL, IADL, FFP, CFS, and falls between the clusters. Linear regression was fitted to test the associations of IC clusters with age and IC index. The correlation between IC measurements or domains was calculated using Spearman's correlation test and visualized by performing a network analysis with the CytoNCA application in Cytoscape (V3.9.1).

A *p*-value of < 0.05 was defined as statistically significant with a two-tailed test. For multiple testing, the *p*-value was adjusted with the Benjamini–Hochberg method.

## Results

### Enrollment and total sample characteristics

Table 1 presents the characteristics of the participants. A total of 909 older inpatients with a mean age of 76.6 ± 9.9 years were included. Among them, 51.4% were male. Decrease in ADL dependency, IADL dependency, physical frailty, and clinical frailty were common in older adults, accounting for 66.7, 62.1, 60.2, and 73.0%, respectively. The three main problems among the IC measurements were physical activities, sleep problems, and impaired vision. Over 60% of patients had a reduction in physical activities, such as walking speed and grip strength; 49.1 and 38.6% of the participants had problems with sleep and vision, respectively. Similar trends were found after adjusting for sex.

**Table 1:** Characteristics of the participants.

**Characteristics**	**Overall**	**Male**	**Female**
*n* (%)	909 (100)	467 (51.3)	442 (40.7)
Age, years, mean ± SD	76.6 ± 9.9	77.4 ± 10.1	76.3 ± 9.8
BMI, kg/m^2^, mean ± SD	23.4 ± 3.9	23.1 ± 3.7	23.6 ± 4.1
BADL score <100, *n* (%)^a^	341 (66.7)	188 (65.5)	153 (68.0)
IADL score <8, *n* (%)^b^	494 (62.1)	219 (56.7)	275 (67.0)
FFP score > 2, *n* (%)^c^	515 (60.2)	278 (63.2)	237 (57.1)
CFS score > 3, *n* (%)^d^	292 (73.0)	155 (73.8)	137 (72.1)
Falls, yes, *n* (%)^e^	205 (22.9)	96 (21.0)	109 (25.1)
**Locomotion**			
SPPB score <10, *n* (%)	577 (63.5)	294 (62.9)	283 (64.0)
Walking speed <1.0 m/s, *n* (%)	717 (78.9)	359 (76.9)	358 (81.0)
Chair rise (5 times) ≥ 12 s, *n* (%)	714 (78.5)	364 (77.9)	350 (79.1)
Grip Strength male <28 kg, female <18 kg, *n* (%)	544 (59.8)	293 (62.7)	251 (56.8)
**Vitality**			
Weight loss > 5%, *n* (%)	138 (15.2)	82 (17.5)	56 (12.7)
BMI <18.5 kg/m^2^, *n* (%)	163 (17.9)	89 (19.6)	74 (16.7)
Eat-10 score ≥3, *n* (%)	120 (13.2)	75 (16.1)	45 (10.2)
**Cognition**			
MMSE score <24, *n* (%)	279 (30.7)	153 (32.8)	126 (28.5)
**Psychological**			
Self-reported depressive symptoms, yes, *n* (%)	159 (17.5)	79 (16.9)	80 (18.1)
AIS, impaired, *n* (%)	446 (49.1)	198 (42.4)	248 (56.1)
Self-reported life satisfaction, not satisfied, *n* (%)	181 (20.2)	85 (18.2)	96 (21.7)
**Sensory**			
Vision, impaired, *n* (%)	351 (38.6)	188 (40.3)	163 (36.9)
Hearing, impaired, *n* (%)	162 (17.8)	98 (21.0)	64 (14.5)

SD, standard deviation; BADL, the Barthel index for basic activities of daily living; IADL, instrumental activities of daily living; FFP, Fried frailty phenotype; CFS, Clinical Frailty Scale; SPPB, Short Physical Performance Battery; Eat-10, 10 Items for Swallowing Assessment; MMSE, Mini-Mental State Examination; AIS, Athens Insomnia Scale.

a−e sample size was 515, 796, 855, 400, and 892 for BADL, IADL, FFP, CFS, and falls, respectively.

### The characteristics of IC domains

**Figure 1:**
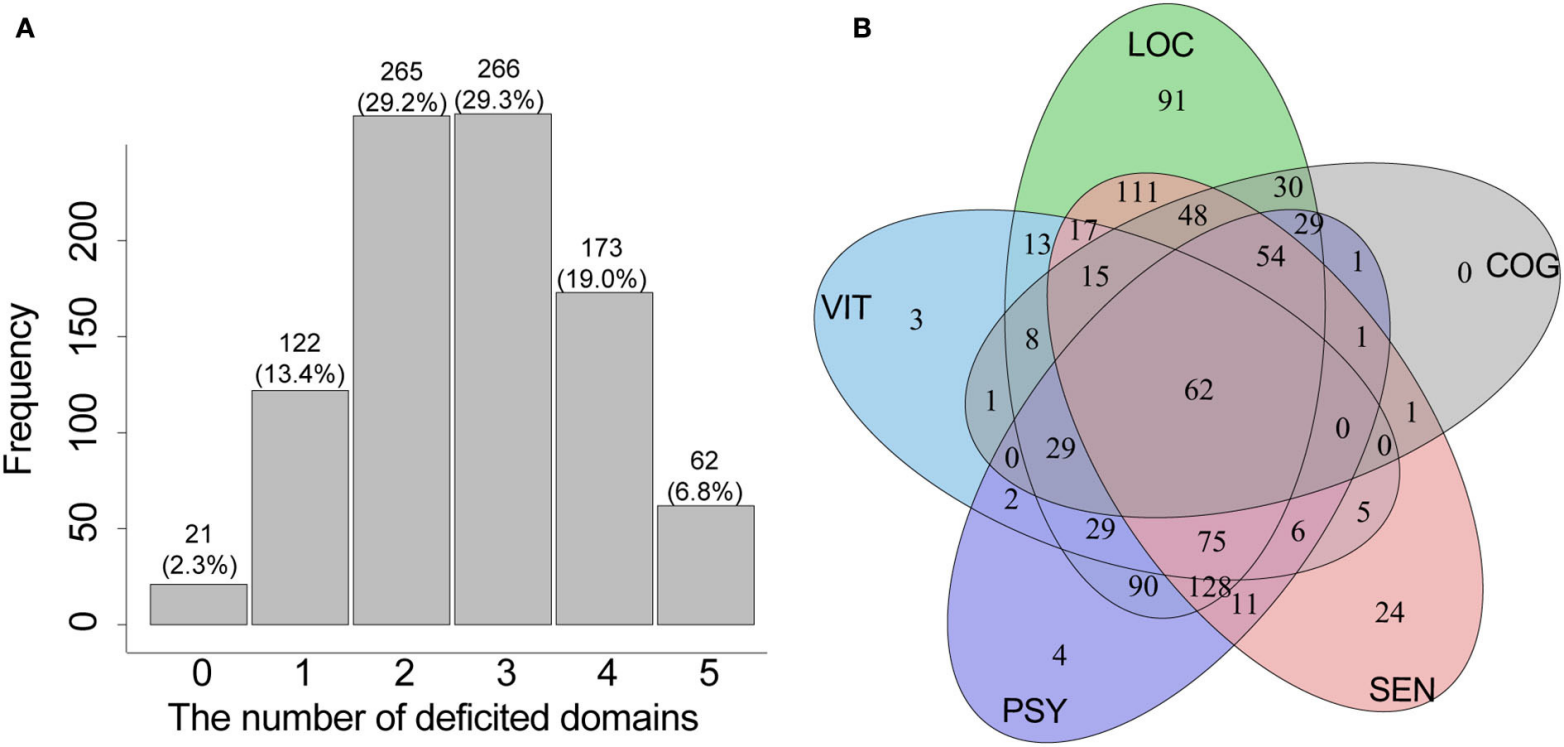
The distribution **(A)** and overlap **(B)** of the IC domains among older inpatients. IC, intrinsic capacity; LOC, locomotion domain; SEN, sensory domain; PSY, psychological domain; COG, cognition domain; VIT, vitality domain.

Figure 1 shows the composition, distribution, and overlap of the IC domains among older patients. The decline of locomotion (about 91.1% of the participants) was the main problem among the five IC domains, followed by sensory impairment (about 61.4% of the participants), psychological impairment (about 57.3% of the participants), cognition decline (30.7%), and vitality problem (29.2%). About 97.7% of the patients had impairment in at least one domain; nearly 7% had deficits in the five domains (Figure 1A). About 29% of the patients had a decline in two or three domains (Figure 1A), and the drop was mainly contributed by co-impairments of locomotion, sensory, and psychological domains (Figure 1B).

### Classification of patients and the characteristics of clusters

**Figure 2:**
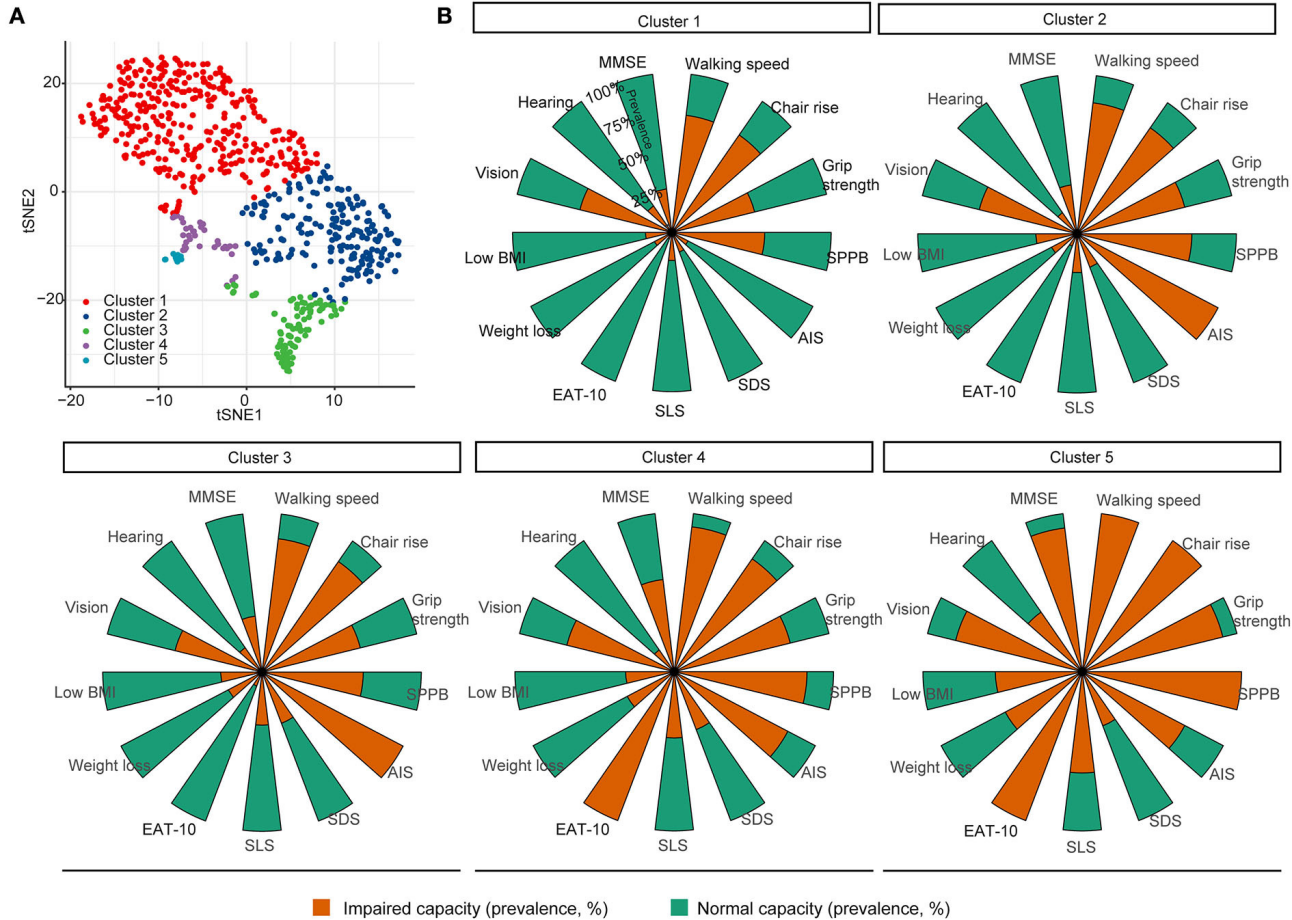
Unsupervised classification of older inpatients based on domains of intrinsic capacity. **(A)** K-means and t-SNE were used to group and visualize the patients, respectively, based on 13 measurements of the intrinsic capacity domains. **(B)** Thirteen measurements depicted the characteristics of each cluster, where the a stacked bar graph showed the prevalence of a deficit. MMSE, Mini-Mental State Examination; SPPB, Short Physical Performance Battery; AIS, Athens Insomnia Scale; SDS, self-reported depressive symptoms; SLS, self-reported life satisfaction; Eat-10, 10 Items for Swallowing Assessment.

A total of five clusters (clusters 1–5) were formed by unsupervised classifications (Figure 2A). Clusters 1–5 could be explained by the loss of IC, with an IC score from 3.32 to 1.87 (Table 2): Cluster 1 may represent patients with a fair to mild decline in intrinsic capacity, while cluster 2 or 3 and cluster 4 or 5 represented patients who had lost a moderate to a great part of IC, respectively. Table 2 also shows how the domains changed from cluster 1 to cluster 5: the capacity index of the locomotion, vitality, and cognition domains significantly reduced from 0.35 to 0.02, from 0.90 to 0.64, and from 0.74 to 0.09, respectively. However, the linear trend for sensory and psychology domains was not observed.

**Table 2:** Intrinsic capacity (IC) and domain scores by clusters among the inpatients.

**Score**	**Cluster 1**	**Cluster 2**	**Cluster 3**	**Cluster 4**	**Cluster 5**	***P* trend**
Locomotion	0.35 (0.35)	0.22 (30.3)	0.26 (0.27)	0.16 (0.32)	0.02 (0.08)	<0.001
Vitality	0.90 (0.19)	0.82 (0.26)	0.81 (0.25)	0.79 (0.26)	0.64 (0.28)	<0.001
Sensory	0.62 (0.31)	0.62 (0.30)	0.62 (0.32)	0.57 (0.32)	0.36 (0.32)	0.126
Psychological	0.71 (0.22)	0.72 (0.19)	0.72 (0.22)	0.69 (0.17)	0.76 (0.26)	0.588
Cognition	0.74 (0.44)	0.70 (0.46)	0.65 (0.48)	0.42 (0.50)	0.09 (0.30)	<0.001
IC	3.32 (0.84)	3.08 (0.90)	3.06 (0.86)	2.61 (0.91)	1.87 (0.66)	<0.001

The clusters may also be represented by different features of IC domains (Figure 2B). The bar graph shows that reduced locomotion, such as reduced walking speed and grip strength, was the main problem among all the patients; the prevalence of impaired locomotion, SLS, weight loss, and low BMI increased from clusters 1–5. Patients in cluster 2 and cluster 3 had a high prevalence of impaired sleep quality. Patients in clusters 4 and 5 had a high prevalence of cognition impairment, swallowing problems, and vision impairment. Patients in cluster 5 showed a high prevalence in hearing impairment. Furthermore, impaired vision was a greater problem in the sensory domain than in the hearing domain; it was present in all clusters but was more prevalent in clusters 4 and 5.

The characteristics of frailty, age, IC index, ADL, and IADL among the five clusters were further investigated and are shown in Table 3. There were 56.6, 11.6, 26.2, 4.3, and 1.3% patients in clusters 1–5, respectively. The patients in clusters 4 and 5 were significantly older than those in other clusters, while the gender difference was not significant among the clusters. Scores of ADL, IADL, FFP, and CFS and the number of falls were significantly changed from clusters 1 to 5; however, the differences between clusters 2 and 3 were not significant for ADL, FFP, CFS, and the prevalence of falls. Taken together, clusters 1–5 were associated with gradually decreased IC scores and functional abilities. In addition, the variation of IC domains was also observed between clusters, indicating heterogeneous trajectories of functional ability in the aging process.

**Table 3:** Association of clusters with frailty, age, sex ADL, IADL, and falls.

	**Cluster 1**	**Cluster 2^#^**	**Cluster 3**	**Cluster 4**	**Cluster 5**	***P* trend**
*N* (%)	473 (56.6)	218 (26.2)	97 (11.6)	36 (4.3)	11 (1.3)	**–**
Age, years, mean (SD)	76.0 (9.4)	76.1 (10.3)	76.2 (9.7)	82.3 (8.5)^*^	85.6 (8.6)^*^	0.005
Sex, male, *n* (%)	262 (55.3)	96 (44.0)	38 (39.1)	20 (55.6)	10 (91.0)	0.195
BADL, median (IQR)	95 (85–100)^**^	90 (70–95)	90 (80–100)	52 (23–72)^**^	5 (1–9)^**^	<0.001
IADL, median (IQR)	7 (5–8)^*^	6 (3–8)	7 (5–8)^*^	1 (1–4)^**^	0 (0–1.5)^**^	<0.001
FFP, median (IQR)	2 (1–3)^**^	2 (1–3)	2 (1–3)	3 (3–4)^**^	4 (3–4.5)^**^	<0.001
CFS, median (IQR)	4 (3–5)^*^	4 (4–6)	4 (4–5)	6 (5–6)^*^	7 (6–7)^*^	<0.001
Falls, yes, *n* (%)	89 (19.0)	53 (24.4)	26 (27.4)	15 (42.8)	2 (18.8)	0.005

SD, standard deviation; IQR, interquartile range; BADL, the Barthel index for basic activities of daily living; IADL, instrumental ADL; FFP, Fried frailty phenotype; CFS, Clinical Frailty Scale.

* *P* adjusted < 0.05;

** *P* adjusted < 0.01;

# Cluster 2 was taken as a reference in comparison between clusters.

### Correlations between measurements and domains of IC

**Figure 3:**
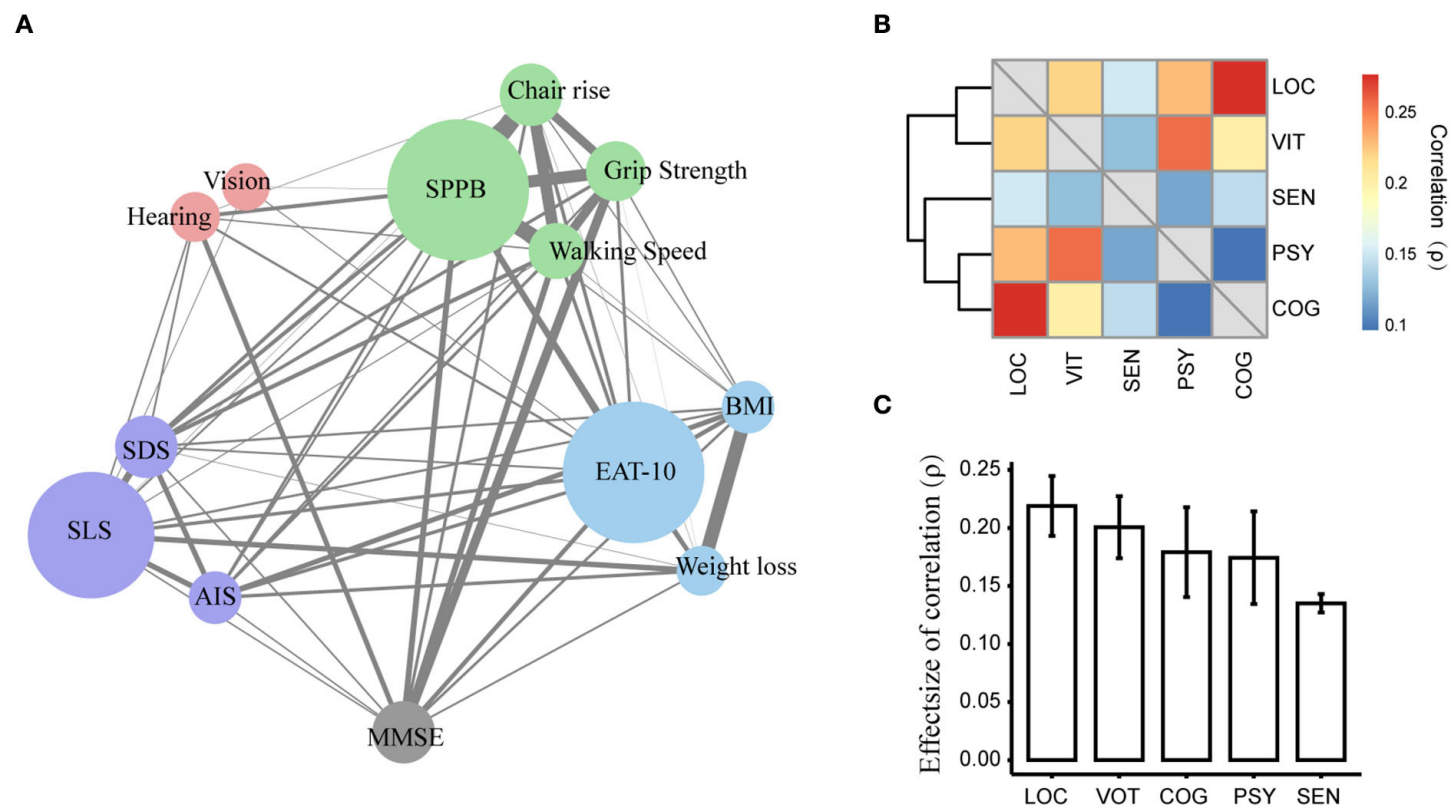
Correlations between the measurement or domains of intrinsic capacity. **(A)** Correlations between measurements. The width of the edges represents the effect size of the correlation; the size of the nodes represents the centrality strength for measurements in the network. Only the significant correlation was plotted as a link; the width of the link represented the effect size of the correlation between the two measurements. **(B)** Correlation between IC domains. Heatmap shows the effect size of the correlations. **(C)** Mean correlation of one domain with others. MMSE, Mini-Mental State Examination; SPPB, Short Physical Performance Battery; AIS, Athens Insomnia Scale; SDS, self-reported depressive symptoms; SLS, self-report life satisfaction; Eat-10, 10 Items for Swallowing Assessment. LOC, locomotion domain; SEN, sensory domain; PSY, psychological domain; COG, cognition domain; VIT, vitality domain.

Measurements in the locomotion domain or the vitality domain showed higher correlations within the domain than with other domains (Figure 3A). For example, the correlation between grip strength and chair rise (effect size ρ = 0.30, *p* < 0.001) was stronger than the correlation between grip strength and lower BMI (effect size ρ = 0.08, *p* = 0.02) (Supplementary material 1). The locomotion domain showed the strongest correlation with the other domains (effect size: ρ = 0.22), indicating that it could be a centralized domain for IC (Figures 3B,C; Supplementary material 2). Furthermore, the Short Physical Performance Battery test and standardized swallowing assessment were the two centralized measurements that closely correlated with most other measurements; vision was only significantly correlated with three nodes (Supplementary material 1).

## Discussion

In the current study, more than 97% of inpatients experienced declines in one or more IC domains, where locomotion impairment was the most prevalent and showed the closest links to other domains. By an unsupervised classification, the inpatients were divided into five clusters significantly associated with age: ADL, IADL, FFP, CFS, and fall. Variations of the IC domains were observed between clusters, indicating heterogeneous trajectories of functional ability in the aging process.

The prevalence of impairment in IC among older hospitalized patients remains unclear, although the degree of IC predicted adverse health outcomes after discharge compared with that at admission (9). The present study showed that more than 90% of the older inpatients showed a decline in at least one IC domain. This finding is similar to that of a previous report of inpatients at Zhejiang Hospital in South China (95%) (9), but it is higher than the prevalence reported by Xuanwu Hospital in North China (69%) (10). The inconsistency could be explained by the fact that some middle-aged adults (aged 50–60 years) were included in the Xuanwu Hospital. It should be noted that the prevalence of the IC decline among hospitalized older patients was at least two times that among older adults living in the community. For example, Locquet et al. and Yu et al. have reported that 25–27% and 4.5–16% of older community dwellers had declined mobility capacity and cognitive capacity, respectively (5, 7); Zeng et al. and our study found between 60 and 91% and about 31% of inpatients had impaired mobility capacity and cognitive capacity, respectively (9). The increasing prevalence reflects progressive declines in individual capacity and the differences in the growth rate between the different domains from community residents to inpatients.

Unsupervised classification of older adults provides a new way to understand the conceptual framework of IC. By interpreting the clusters in the present work, we found two potential modes in the trajectories of the aging process. The first trajectory indicates a gradually decreased IC score, where it was considered a whole indicator, and the clusters were significantly associated with functional ability such as ADL. The second indicates the heterogeneity among aged people. For example, clusters 1–3 showed similar aged adults, but sleep problems were more prevalent in clusters 2 and 3, which suggest that sleep problems in the psychological domain contributed much to the classification. However, there are some limitations in the clustering analysis. The number of potential clusters or modes of IC among older adults remain uncertain. Whether we should interpret the clusters by domains or by measurements of IC needs to be further discussed. The WHO proposed a policy framework that identified three subgroups of older adults: those with relatively high and stable capacity, decreasing capacity, and substantial losses in intrinsic capacity (29). In this study, five clusters of inpatients were identified in this work, while and clusters 4 and 3 comprised community residents without and with long-term care, respectively (6, 30). Although different studies showed different numbers of clusters, one commonality exists: all clusters could score functional ability from high to low. For example, our results showed significant linear associations of the clusters with increased age, declined functional independence, number of falls, and frailty; the latter classified the older adults into high IC/robust cluster, intermediate IC/prefrail cluster, and low IC/prefrail–frail cluster (6). Another shared commonality in the clusters is that subgroups of the older adults based on IC would help discover the special patterns of their needs when preventions or interventions are necessary. For example, a sensory dysfunction subgroup was identified in older community dwellers with long-term care. The elderly in this cluster experienced high rates of adverse functional outcomes (30), implying that early detection of sensory impairment might provide an opportunity to prevent functional decline (31).

In the present study, about 56% of the inpatients could be identified as a relatively normal stable class (cluster 1), <10% of the inpatients belonged to the all-dysfunction class (clusters 4 and 5), and ~38% of the inpatients (clusters 1 and 2) had a high proportion of sleep problems. In our study, two domain foci were addressed: (1) A high prevalence of locomotion impairment was observed in all clusters, and it gradually increased from clusters 1 to 5. A high prevalence of locomotion dysfunction (90%) was observed not only in the hospitalized older adults but also in the older adults living in the community (4, 6, 32). These results indicate that locomotion capacity might decrease faster than other capacities at the upstream location of trajectories in the healthy aging process; maintaining or recovering locomotion ability should be advised to most of the population. (2) Sleep problems may commonly exist in certain groups of older inpatients. Clinicians should pay more attention to these people not only because sleep problems affect their quality of life (33) but also because acute sleep deprivation easily happens during the period of hospitalization for older adults (34). Locomotion capacity showed strong correlations with the other capacities in this study. Similarly, physical activity programs positively affected various aspects of sleep and other health outcomes in the older adults (35–37). Consequently, promoting physical activity to recover, maintain, and even improve IC according to classifications could be one of the most essential strategies for older inpatients.

There is no single assessment tool to determine IC. As a result, the measurement of vitality showed significant heterogeneity. According to the WHO consortium, vitality was proposed to be measured by using the Mini-Nutritional Assessment (MNA) (38). The ICOPE app is evaluated by recording weight and appetite loss (39)—the two most commonly used measurements. Vitality is defined through the concept of energy balance, and some measurements reflecting the metabolic modifications were suggested. Cesari et al. suggested that hormonal and cardiorespiratory fitness, which affect energy metabolism, should be taken into account in this domain (40). Other measurements, such as BMI, abdominal circumference, and mid-upper arm circumference, were also used to measure altered metabolic status since changes in body composition occur with aging (4, 41). In the present study, we used three measurements (BMI, weight loss, and standardized swallowing assessment) to estimate vitality. It should be noted that handgrip strength was not used to represent vitality but was used in the measuring locomotion capacity in our study. We did not follow many previous studies that showed handgrip strength was associated with malnutrition and can indirectly reflect vitality capacity (1, 41, 42). We assume that handgrip strength was closely related to locomotion-related measurements and therefore should be used to test locomotion capacity. Hence, the present study proved our hypothesis that the correlation of handgrip strength with SPPB, chair rise, and walking speed was stronger than that with vitality measurements (BMI, weight loss, and swallowing impairment). Whether handgrip strength should be used to measure locomotion capacity or vitality capacity is not yet clear; more strong evidence should be provided by cohort studies. Furthermore, we suggest that measurements such as cardiorespiratory fitness and biomarkers (insulin-like growth factor) should not be generalized to all older adults because they are time-consuming, labor-intensive, and expensive, particularly for low- and middle-income countries.

In the present study, we found some measurements in the same IC domain were not closely correlated. For example, hearing and vision were not significantly associated, but they were analyzed as a whole. These results raised concerns that whether these measurements could be used as indicators of sensory impairments, and it could affect the way we use the sensory domain in clinical practices. Although the WHO and some previous studies pointed out that both hearing and vision impairments belong to sensory problems (2, 8, 13), there is no consensus on whether we should use them as one or two domains. Our results indicated that they should be used separately in clinical practice because not only they represent different physiopathologies but also they had a great variation of links with other measurements or domains and contributed differently to the clusters. Similar concerns were raised regarding the vitality domain, which was measured by Eat-10, BMI, and weight loss. The link between BMI and Eat-10 was not as strong as that between BMI and weight loss. We added Eat-10 as an indicator of vitality based on the hypothesis that it could affect energy intake. The extent to which the measurements should be related before they can be integrated together to measure one domain remains unclear. Our results showed swallowing problem was prevalent in clusters 4 and 5, indicating it could be a predictor of reduced functional ability in older adults. However, using Eat-10 as a measurement of vitality should be further validated.

The main strengths of our study are as follows: First, this is the most extensive study that systematically assessed the primary characteristics of IC among older inpatients. In addition to assessing the primary features of IC, we classified the inpatients into several clusters, which gave a better understanding of the conceptual framework of IC. Based on the clusters, we observed not only a gradual decrease in IC with aging (e.g., the linear association of clusters 1–5 with age and IC index) but also the variation in IC trajectories or functional abilities among different individuals (e.g., clusters 1–3 showed similar age, but they were significantly different in domains of IC). Second, the GCA team was trained and organized professionally, guaranteeing a higher measurement validity.

Nonetheless, this study had some limitations. First, the data were obtained from only one hospital, reducing the generalizability of our results to overall inpatients of China. Although we obtained results similar to a previous study that was also performed in a Chinese hospital, high variation was observed between different geriatric regions among community residents (4). Second, there might be a selection bias in sampling. We excluded patients with incomplete measurements of IC. It is probable that these patients were unwilling to participate or were unable to undergo physical or mental tests due to their diseases for which they visited the hospital. Future studies could investigate this possibility by comparing the differences of some domains between the included and the excluded inpatients in several domains.

Last, we identified subgroups of older inpatients and assessed associations between the clusters and functional abilities such as ADL and frailty. However, further cohort studies are necessary to prove whether the clusters can predict health outcomes and how to integrate them into healthcare need.

## Conclusion

Significant declines were observed in IC among the hospitalized older adults, and disparities existed between the domains of IC among these patients. In this study, the older inpatients were classified into different subgroups based on the measurements of IC, and the clusters were significantly associated with increased age, ADL dependence, frailty, and number of falls. The classification and the primary assessment of IC enabled us to find different patterns of decline in functional abilities among hospitalized patients. This finding is pivotal for designing integrated treatment or care models in clinical practice.

## Data availability statement

The original contributions presented in the study are included in the article/Supplementary material, further inquiries can be directed to the corresponding author/s.

## Ethics statement

The studies involving human participants were reviewed and approved by the Medical Ethics Committee of Beijing Hospital. The patients/participants provided their written informed consent to participate in this study.

## Author contributions

QZ and LS contributed to conceptualization and methodology. JS and JL contributed to data collection. JZ and HL participated in functional assessment. WW and QZ analyzed the data and wrote the original draft. All authors contributed to implement and revise the manuscript.

## Funding

This work was supported in whole, or in part, by the National High Level Hospital Clinical Research Funding (BJ-2022-181) and China National Key R&D Program (2020YFC2009006 and 2020YFC2009000).

## Conflict of interest

The authors declare that the research was conducted in the absence of any commercial or financial relationships that could be construed as a potential conflict of interest.

## Publisher's note

All claims expressed in this article are solely those of the authors and do not necessarily represent those of their affiliated organizations, or those of the publisher, the editors and the reviewers. Any product that may be evaluated in this article, or claim that may be made by its manufacturer, is not guaranteed or endorsed by the publisher.
