# Supplementary material for: Approaching person-centered clinical practice: A cluster analysis of older inpatients utilizing the measurements of intrinsic capacity
Source: Front Public Health. 2022 Nov 11;10:1045421. doi: 10.3389/fpubh.2022.1045421 (PMC9692078; doi:10.3389/fpubh.2022.1045421)
Supplement: Supplementary file 1 [file Data_Sheet_1.DOCX]

**Supplementary file 1** Correlations between the measurements of intrinsic capacity

| Measurement | Measurement | Effect size (ρ) | *P* value | Adjusted *p* value |
| --- | --- | --- | --- | --- |
| walking speed | chair rise | 0.386 | <0.001 | <0.001 |
| walking speed | grip strength | 0.318 | <0.001 | <0.001 |
| walking speed | BMI | 0.094 | 0.004 | 0.006 |
| walking speed | SDS | 0.103 | 0.002 | 0.002 |
| walking speed | hearing | 0.114 | 0.001 | 0.001 |
| walking speed | MMSE | 0.233 | <0.001 | <0.001 |
| walking speed | EAT-10 | 0.165 | <0.001 | <0.001 |
| walking speed | AIS | 0.156 | <0.001 | <0.001 |
| walking speed | SLS | 0.182 | <0.001 | <0.001 |
| walking speed | SPPB | 0.484 | <0.001 | <0.001 |
| chair rise | grip strength | 0.304 | <0.001 | <0.001 |
| chair rise | weight loss | 0.086 | 0.009 | 0.011 |
| chair rise | BMI | 0.111 | 0.001 | 0.001 |
| chair rise | SDS | 0.085 | 0.010 | 0.012 |
| chair rise | hearing | 0.089 | 0.007 | 0.009 |
| chair rise | MMSE | 0.156 | <0.001 | <0.001 |
| chair rise | EAT-10 | 0.163 | <0.001 | <0.001 |
| chair rise | AIS | 0.133 | <0.001 | <0.001 |
| chair rise | SLS | 0.167 | <0.001 | <0.001 |
| chair rise | SPPB | 0.624 | <0.001 | <0.001 |
| grip strength | weight loss | 0.077 | 0.019 | 0.022 |
| grip strength | BMI | 0.113 | 0.001 | 0.001 |
| grip strength | hearing | 0.176 | <0.001 | <0.001 |
| grip strength | MMSE | 0.316 | <0.001 | <0.001 |
| grip strength | EAT-10 | 0.157 | <0.001 | <0.001 |
| grip strength | AIS | 0.133 | <0.001 | <0.001 |
| grip strength | SLS | 0.161 | <0.001 | <0.001 |
| grip strength | SPPB | 0.394 | <0.001 | <0.001 |
| weight loss | BMI | 0.433 | <0.001 | <0.001 |
| weight loss | SDS | 0.216 | <0.001 | <0.001 |
| weight loss | MMSE | 0.123 | <0.001 | <0.001 |
| weight loss | EAT-10 | 0.197 | <0.001 | <0.001 |
| weight loss | AIS | 0.167 | <0.001 | <0.001 |
| weight loss | SLS | 0.091 | 0.006 | 0.008 |
| weight loss | SPPB | 0.133 | <0.001 | <0.001 |
| BMI | SDS | 0.139 | <0.001 | <0.001 |
| BMI | MMSE | 0.142 | <0.001 | <0.001 |
| BMI | EAT-10 | 0.187 | <0.001 | <0.001 |
| BMI | AIS | 0.207 | <0.001 | <0.001 |
| BMI | SLS | 0.138 | <0.001 | <0.001 |
| BMI | SPPB | 0.107 | 0.001 | 0.002 |
| SDS | vision | 0.098 | 0.003 | 0.004 |
| SDS | hearing | 0.119 | <0.001 | <0.001 |
| SDS | MMSE | 0.114 | 0.001 | 0.001 |
| SDS | EAT-10 | 0.165 | <0.001 | <0.001 |
| SDS | AIS | 0.219 | <0.001 | <0.001 |
| SDS | SLS | 0.256 | <0.001 | <0.001 |
| SDS | SPPB | 0.124 | <0.001 | <0.001 |
| vision | EAT-10 | 0.106 | <0.001 | <0.001 |
| vision | SPPB | 0.087 | 0.009 | 0.011 |
| hearing | MMSE | 0.207 | <0.001 | <0.001 |
| hearing | EAT-10 | 0.136 | <0.001 | <0.001 |
| hearing | SLS | 0.125 | <0.001 | <0.001 |
| hearing | SPPB | 0.127 | <0.001 | <0.001 |
| MMSE | EAT-10 | 0.189 | <0.001 | <0.001 |
| MMSE | AIS | 0.112 | 0.001 | 0.001 |
| MMSE | SLS | 0.119 | <0.001 | 0.001 |
| MMSE | SPPB | 0.227 | <0.001 | <0.001 |
| EAT-10 | AIS | 0.166 | <0.001 | <0.001 |
| EAT-10 | SLS | 0.128 | <0.001 | <0.001 |
| EAT-10 | SPPB | 0.233 | <0.001 | <0.001 |
| AIS | SLS | 0.210 | <0.001 | <0.001 |
| AIS | SPPB | 0.146 | <0.001 | <0.001 |
| SLS | SPPB | 0.180 | <0.001 | <0.001 |

MMSE, mini-mental state examination; SPPB, short physical performance battery; AIS, Athens insomnia scale; SDS, self-reported depressive symptoms; SLS, self-report life satisfaction; Eat-10, ten items for swallowing assessment.

**Supplementary file 2** Correlations between the domains of intrinsic capacity

| Domain | Domain | Effect size (ρ) | P value | Adjusted p value |
| --- | --- | --- | --- | --- |
| Locomotion | Vitality | 0.217 | <0.001 | <0.001 |
| Locomotion | Sensory | 0.151 | <0.001 | <0.001 |
| Locomotion | Psychological | 0.229 | <0.001 | <0.001 |
| Locomotion | Cognition | 0.276 | <0.001 | <0.001 |
| Vitality | Sensory | 0.128 | 0.001 | <0.001 |
| Vitality | Psychological | 0.256 | <0.001 | <0.001 |
| Vitality | Cognition | 0.200 | <0.001 | <0.001 |
| Sensory | Psychological | 0.116 | 0.002 | 0.002 |
| Sensory | Cognition | 0.144 | <0.001 | <0.001 |
| Psychological | Cognition | 0.096 | 0.011 | 0.011 |
